# Response: “Letter to the Editor: Lessons to Be Learned From the COVID-19 Pandemic: Some Further Ideas”

**DOI:** 10.3389/ijph.2025.1609398

**Published:** 2026-02-02

**Authors:** Gerry A. Quinn, Ronan Connolly, Coilín ÓhAiseadha, Paul Hynds, Philipp Bagus, Ronald B. Brown, Carlos F. Cáceres, Clare Craig, Jose L. Domingo, Norman Fenton, Paul Frijters, Steven Hatfill, Raymond Heymans, Ari R. Joffe, Rosamond Jones, Gordan Lauc, Alan Mordue, Greta Mushet, Anton O’Connor, Jane Orient, José Antonio Peña-Ramos, Harvey A. Risch, Jessica Rose, Antonio Sánchez-Bayón, Ricardo F. Savaris, Michaéla C. Schippers, Dragos Simandan, Willie Soon, Yaffa Shir-Raz, Demetrios A. Spandidos, Beny Spira, Aristides M. Tsatsakis, Harald Walach

**Affiliations:** 1 Centre for Molecular Biosciences, Ulster University, Coleraine, United Kingdom; 2 Center for Environmental Research and Earth Sciences (CERES), Salem, MA, United States; 3 Independent Researcher, Dublin, Ireland; 4 Department of Public Health, Health Service Executive, Dublin, Ireland; 5 Sustainability and Health Research Hub, Technological University Dublin, Dublin, Ireland; 6 Irish Centre for Research in Applied Geosciences, School of Earth Sciences, College of Science, University College Dublin, Cork, Ireland; 7 Department of Applied Economics, Faculty of Law and Social Sciences, Rey Juan Carlos University, Móstoles, Spain; 8 School of Public Health Sciences, Faculty of Applied Health Sciences, University of Waterloo, Waterloo, ON, Canada; 9 School of Public Health and Administration, Universidad Peruana Cayetano Heredia, Miraflores, Peru; 10 Health Advisory and Recovery Team, London, United Kingdom; 11 Faculty of Medicine and Health Sciences, University of Rovira i Virgili, Reus/Tarragona, Spain; 12 School of Electronic Engineering and Computer Science, Queen Mary University, London, United Kingdom; 13 Department of Social Policy, London School of Economics, London, United Kingdom; 14 London Center For Policy Research, New York, NY, United States; 15 Independent Researcher, Koedijk, Netherlands; 16 John Dossetor Health Ethics Center, University of Alberta, Edmonton, AB, Canada; 17 Faculty of Pharmacy and Biochemistry, University of Zagreb, Zagreb, Croatia; 18 Independent Researcher, Melrose, United Kingdom; 19 Association of American Physicians and Surgeons, Tucson, AZ, United States; 20 Department of Political Science and Administration, University of Granada, Granada, Spain; 21 School of Public Health, Yale University, New Haven, CT, United States; 22 Brownstone Institute, Austin, TX, United States; 23 Department of Applied Economics, Rey Juan Carlos University, Móstoles, Spain; 24 Department of Gynecology and Obstetrics, Faculty of Medicine, Federal University of Rio Grande do Sul, Porto Alegre, Brazil; 25 Department of Organisation and Personnel Management, Rotterdam School of Management, Erasmus University Rotterdam, Rotterdam, Netherlands; 26 Faculty of Social Sciences, Brock University, St. Catharines, ON, Canada; 27 Department of Earth Sciences, Institute of Earth Physics and Space Science, Sopron, Hungary; 28 School of Public Health, University of Haifa, Haifa, Israel; 29 Laboratory of Clinical Virology, School of Medicine, University of Crete, Heraklion, Greece; 30 Departamento de Microbiologia, Instituto de Ciências Biomédicas, Universidade de São Paulo, São Paulo, Brazil; 31 Center of Toxicology Science & Applications, Medical School, University of Crete, Heraklion, Greece; 32 Universidad Ecotec, Samborondón, Ecuador; 33 Sechenov IM First State Medical University, Moscow, Russia; 34 Next Society Institute, Kazimieras Simonavičius University, Vilnius, Lithuania

**Keywords:** all cause mortality, government stringency independence, non-pharmaceutical interventions, pandemic management lessons, vaccine induced myocarditis

We thank the author of the Letter to the Editor for their positive feedback on our manuscript “What Lessons can Be Learned From the Management of the COVID-19 Pandemic?” and for providing additional suggestions to strengthen the discussion. The author highlights a number of points that broadly dovetail with our original assessment, however, given the contentious nature of many of the statements discussed, we believe it is important to be careful in describing precisely what is known from the published literature so far. Given the evolving nature of what has been published over time, we expect that many of the conclusions we reached in our original manuscript, as well as in this reply will transpire to have been just been scratching the surface. Nonetheless we think it’s important to accurately document what is already known from current peer reviewed literature. Therefore, we welcome this opportunity to engage in a constructive dialogue and to clarify some of the points we made which may not have been fully understood by some of our readers. 

Given the vast amount of peer-reviewed literature generated on COVID-19, there was not always space in our manuscript [1] to expand upon each point that we made. Even so, our manuscript is still five times the recommended length of any article in IJPH and included four times the usual number of references, so we are grateful to the Editors for allocating so much space to this important topic.

On the independence of pandemic progression from government measures (page 8):

We realize that correlation does not necessarily mean causation. However, just to clarify the author of the Letter to the Editors’ point, we presume that they are referring to the positive but unfavourable correlation between excess deaths and mortality. Furthermore, we presume the author is referring to the Stringency Index from the Oxford COVID-19 Government Response Tracker (OxCGRT), as no standalone “lockdown index” database exists, but this is a common shorthand [1].

It is very hard to say whether measures were productive or counter-productive, because attempts to assess the impacts of government measures on all-cause mortality are complicated by factors that may have acted as confounders or random drivers of mortality in one direction or another. As Oh et al. [2] have pointed out, the collateral effects of the pandemic may be associated with mortality risk through various pathways.

Given this, we also caution that the Oxford team has emphasized in their publications that “stronger and more timely government responses were crucial in curbing the spread,” highlighting a contrast with some data-derived interpretations. Given these clearly differing perspectives, we suggest that further critical and open-minded research into this controversial topic is warranted.

Nonetheless, we echo the general thrust of the authors general point here, for example, in our manuscript [3], we critiqued the evidence for the effectiveness of NPIs by stating: “assessments that were not solely based on counterfactual scenarios often found that the progression of the pandemic was largely independent of government measures.” As research by Herby et al. [4, 5] points out, stricter measures (NPIs) were associated with very little change in COVID-19 deaths when examined by meta-analysing “difference in difference” studies. This is similar to the data presented in the Letter to the Editor. Another meta-analysis also found high quality evidence demonstrating the lack of an effect of NPIs on any outcome [6].

Additionally, we have pointed to instances where these measures had, on cost-benefit analysis, clearly harmful consequences [7–14].

One underappreciated factor concerns the loss of purpose and meaning experienced by many individuals during prolonged lockdowns and social restrictions. Such “purpose deprivation” has been shown to be related to declines in mental health, motivation, and social cohesion. Empirical work on *life crafting* interventions suggests that actively reflecting on and articulating one’s personal goals and values can help restore a sense of agency and meaning [15]. More recently, scalable initiatives such as the *Letters to the Future Challenge* [16] suggest that purpose-oriented writing tasks can engage students and citizens in envisioning positive futures and aligning personal goals with collective challenges.

These findings reinforce our original call [3] for greater empirical scrutiny over models, suggesting lessons like prioritizing healthcare resilience and cost-benefit analyses before considering any future use of NPIs.

On the incidence of transient myocarditis and/or pericarditis (page 15):

Regarding the terminology for myocarditis and pericarditis. While we are highly concerned about rates of cardiovascular events, we stand by our specific use of the term ‘uncommon’ in this case. This section addresses high-sensitivity observational studies from vaccination programmes within individual institutions, which indicated that cardiovascular symptoms after the second dose are “very common”. However, it is critical to distinguish between subjective symptoms and a confirmed diagnosis. The three references we provided specifically support our claim that the incidence of clinically confirmed myo- or pericarditis is ‘uncommon’. In the paper by Mansanguan et al., 7 students or 2.33% exhibited at least one elevated cardiac biomarker or positive lab assessment; however, only one was confirmed as clinical myopericarditis [17].

Similarly, in the study by Chiu et al. [18], only one person was diagnosed with mild clinical myocarditis. As the authors state: “cardiac symptoms are common after the second dose of BNT162b2 vaccine, but the incidences of significant arrhythmias and myocarditis are only 0.1%,” although the study notes that 0.13% had mild myocarditis, an incidence greater than 0.1%, which again is ‘uncommon’. However, we note that 17.1% of students in the study had at least one cardiac symptom after the second vaccine dose, mostly chest pain and palpitations [18]. Finally, in the Buergin et al. study, vaccine-associated myocardial injury was adjudicated in 22 participants (2.8% [95% CI 1.7%–4.3%]), but these were not confirmed as clinical myopericarditis [19].

Still, we are extremely concerned about the mention of laboratory indicators of subclinical or transient heart damage in some of these manuscripts, since the heart has very limited abilities to repair itself after damage. Recent follow-up studies after administration of mRNA based covid vaccines have shown that despite inflammatory and cardiac biomarkers returning to normal ranges, one-third of patients who had been admitted for clinical myocarditis continue to experience symptoms. This highlights the need for long-term follow-up studies [20]. This concern aligns with our manuscript’s emphasis on a thorough and transparent assessment of vaccine-related risks. In addition, the incidence of myocarditis in young males after COVID-19 vaccine of approximately 1/5000 may be uncommon, but at a population level is highly significant [21].

Some of our authors, who are also MDs, have also added some background to this subject stating that myocarditis is a difficult diagnosis to make and unless clinicians are suspicious it can easily be mistaken for other heart diseases particularly in older people. However, it is wrong to consider hospital diagnosed myo/pericarditis as the only harmful outcome. There are multiple other adverse outcomes many of which have been poorly measured. Furthermore, even in terms of cardiac events, the heart damage seen in teenage boys after a booster with 29% being symptomatic as well as 3% having raised troponin and seen in middle aged males and females among university staff indicates that the myocarditis that reaches the clinical threshold for diagnosis is only the tip of an iceberg [17, 18]. They too are very concerned about the implications of this greater number of people with subclinical changes in their heart and regret the missed opportunities to fully investigate this.

On all-cause mortality comparisons between vaccinated and unvaccinated groups (page 16):

Regarding all-cause mortality comparisons between vaccinated and unvaccinated groups, we agree with the comments in the Letter to the Editor. The ONS data show that, over time, the death risk for people with 1–2 vaccine doses rose steadily and, in several age groups (18–39, 80–89, 90+), crossed above the unvaccinated level, for both total deaths and non-COVID deaths. This regression analysis makes the pattern clear and predictable. We regret that ONS ceased detailed monthly reporting after data for May 2023 (final release August 2023), preventing verification of projected crossovers in 2024. Furthermore some analyses, such as that by Fenton et al. [22], suggest that the ONS data may have itself underestimated mortality in the vaccinated cohort, which, if true, would make the observed trend even more concerning.

**FIGURE 1:**
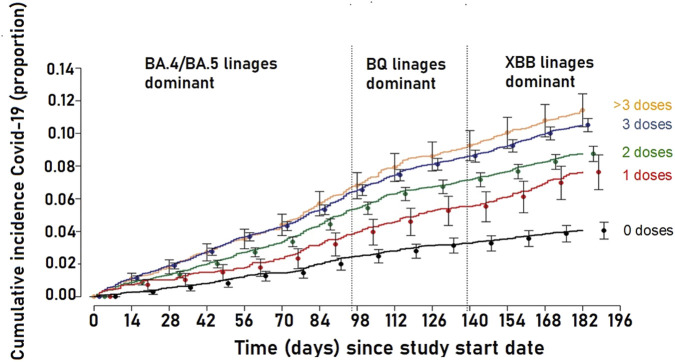
Cumulative incidence of coronavirus disease 2019 (COVID-19) for study participants stratified by the number of COVID-19 vaccine doses previously received. Study period was 12 September 2022 to 27 March 2023, Cleveland USA. Point estimates and 95% confidence intervals are jittered along the x-axis to improve visibility. Adapted from “Effectiveness of the Coronavirus Disease 2019 Bivalent Vaccine” Shrestha et al. [22] Content covered by Crown Copyright (not required to obtain permission to reuse this content).

On vaccines preventing COVID-19 infection and transmission (page 17):

With respect to vaccines preventing COVID-19 infection and transmission, we appreciate the comment on this issue. Indeed, we note the study by Shrestha et al. [23], that suggests the addition of multiple vaccines increased the risk of catching COVID-19 (Figure 1) i.e., the opposite of what should be expected from an effective vaccine.

We appreciate these further comments on our manuscript, which we believe not only reinforce the lessons we outlined in our manuscript but also underscore the critical and ongoing need for a rigorous, evidence-based reassessment of pandemic management strategies.
